# Novel Mutations in KCNJ10 Gene Associated With SeSAME Syndrome: Rare Disorder With Possible Common Mutation

**DOI:** 10.1002/mgg3.70194

**Published:** 2026-02-22

**Authors:** Shayan Shakeri, Sanaz Mohammadi, Forough Sadeghipour, Marjan Masoudi, Mona Entezam

**Affiliations:** ^1^ Department of Medical Genetics, School of Medicine Shiraz University of Medical Sciences Shiraz Iran; ^2^ Comprehensive Medical Genetic Center Shiraz University of Medical Sciences Shiraz Iran

**Keywords:** EAST syndrome, KCNJ10, SeSAME syndrome, whole‐exome sequencing (WES)

## Abstract

**Background:**

Mutations in the *KCNJ10* gene cause SeSAME syndrome, an autosomal recessive disorder characterised by seizures, sensorineural deafness, ataxia, intellectual impairment and electrolyte imbalances. *KCNJ10* encodes an inwardly rectifying potassium channel Kir4.1, which is essential for preserving potassium ion homeostasis.

**Methods:**

We assessed three Iranian families with SeSAME syndrome–like symptoms through whole‐exome sequencing (WES). Segregation analysis and Sanger sequencing were also used to confirm identified mutations. Additionally, bioinformatic tools were utilised to predict the pathogenicity of the variants.

**Results:**

We identified two novel *KCNJ10* mutations, c.967 T>C (p.Y323H) and c.352G>A (p.A118T) in three families. While there was no evidence of renal involvement, the probands from these families displayed early‐onset seizures, ataxia, developmental delays and hearing abnormalities. Based on the Kir4.1 protein's structural modelling, the stability of the channel is influenced by both mutations. Precisely, p.A118T alters the transmembrane domain that is critical to channel operation, whereas p.Y323H modifies the cytoplasmic C‐terminal domain, which may compromise intracellular localisation and regulation.

**Conclusion:**

Our findings can expand the spectrum of mutations in the *KCNJ10* gene and provide insight into the genotype–phenotype correlation in the SeSAME syndrome.

## Graphical abstract

Whole‐exome sequencing in three Iranian families identified two novel KCNJ10 variants (p.A118T, p.Y323H) associated with seizures, ataxia, developmental delay and hearing loss without renal involvement.

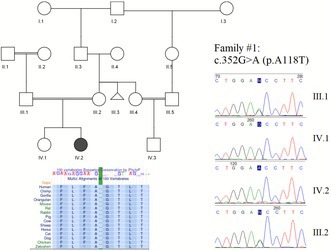

## 1 Introduction

The *KCNJ10* gene (OMIM:602208), located at 1q23.2, encodes the Kir4.1 protein, an inwardly rectifying potassium channel (subfamily J, member 10). Kir4.1 is highly expressed in the central nervous system's renal epithelial cells, inner ear cells and glial cells. Potassium (K^+^) channels are critical components of the mammalian system, playing essential roles in regulating electrical activity at the membrane, maintaining membrane potential and controlling the passage of potassium ions across cell membranes. These channels are integral to fluid balance, cellular signalling and neuronal excitability. Mutations in the *KCNJ10* gene can impair both homomeric Kir4.1 (Bockenhauer et al. 2009) and heteromeric Kir4.1/Kir5.1 (Reichold et al. 2010) channels. Kir4.1 channels are particularly important for maintaining potassium (K^+^) homeostasis in the human brain, as seizures are a primary clinical manifestation of homomeric Kir4.1 loss‐of‐function mutations (Bockenhauer et al. 2009; Scholl et al. 2009).

SeSAME (Epilepsy, Ataxia, Sensorineural Deafness and Tubulopathy) syndrome, also known as EAST syndrome, is a rare autosomal recessive disorder associated with mutations in the *KCNJ10* gene, which was first identified in 2009. Since then, additional mutations in this gene have been linked to various clinical manifestations (Scholl et al. 2009). This syndrome is characterised by a constellation of symptoms, including seizures, sensorineural deafness, ataxia, intellectual disability and electrolyte imbalances, most notably hypokalaemia (Bockenhauer et al. 2009). Intellectual impairment may only sometimes be present, depending on the nature and location of the mutations. The disease exhibits a broad spectrum of clinical signs (Abdelhadi et al. 2016). A study also reported that *KCNJ10* variants were enriched in paroxysmal kinesigenic dyskinesia (PKD) disease cohort (Huang et al. 2024). Additionally, heterozygous variants in KCNJ10 have been reported to associate with recurrent and transient attacks of unilateral or bilateral dystonia and/or choreoathetosis (Wirth et al. 2024).

This study presents the genetic evaluation of three unrelated Iranian families with SeSAME/EAST syndrome in which two novel mutations in the *KCNJ10* gene were identified by Whole Exome Sequencing (WES) analysis.

## 2 Methods and Patients

### 2.1 Patients

Three unrelated Iranian families were referred to the Shiraz Comprehensive Medical Genetics Center for genetic evaluation due to clinical presentations, including developmental delay, seizures and neurological abnormalities. All families were consanguineous. Genetic counselling was provided to all families before proceeding with the evaluation. Written informed consent was obtained from all adult participants and the parents of minor patients. The local Ethics Committee approved the study.

### 2.2 Whole Exome Sequencing

Whole‐exome sequencing was conducted on the three probands using the Illumina HiSeq4000 platform. The sequencing reads were aligned to the hg38 reference genome using BWA. Variants were called using HaplotypeCaller (GATK) and annotated through ANNOVAR. A rigorous filtering pipeline was applied to prioritise pathogenic variants: (a) Exclusion Criteria: intronic variations, synonymous variants without splicing impact (With the help of SpliceAI tool with the score cutoff of 0.2 (de Sainte Agathe et al. 2023)) and Variants with a frequency > 1% in population databases, including GnomAD v.4, 1000 Genomes Project phase 3 and Iranome v.1. (b) Pathogenicity prediction tools were used, including PolyPhen‐2 (Adzhubei et al. 2010), SIFT (Ng and Henikoff 2001), Mutation Taster (Schwarz et al. 2014), MetaRNN (Li et al. 2022) (a recurrent neural network‐based ensemble score integrating 24 prediction algorithms and allele frequency information) and CADD (Rentzsch et al. 2019) to investigate the pathogenicity of the identified missense variants. This was done by a professional genomic variant curator according to the tools developers’ declared cutoffs. Additionally, PhyloP scores (Siepel et al. 2005) were used to determine the conservation of the affected residue.

### 2.3 Sanger Sequencing and Segregation Analysis

DNA was extracted from whole blood samples of the proband, parents and other affected family members using the FAVORGEN Blood Genomic DNA Extraction Mini Kit (Taiwan). Specific primers were designed for each identified variant and PCR followed by Sanger sequencing was performed for mutation confirmation along with the segregation analysis of appropriate individuals at each pedigree.

### 2.4 Homology Modeling

Using accession number P78508, the amino acid sequence of *KCNJ10* was obtained from the UniProt database. Phyre2 was used to simulate the human wild‐type (WT) and mutant Kir4.1 structures in monomeric form. The highest‐ranking predicted structures were refined using ModRefiner to remove residues in prohibited areas and perform energy reduction of structures. The rat Kir4.1 channel protein sequence (which shares 98% amino acid identity with human Kir4.1) in complex with the phospholipid phosphatidylinositol 4,5‐bisphosphate (PIP2) (PDB:8i5m) was downloaded from the Protein Data Bank and used as the template. The final homotetrameric structure of Kir4.1 was produced by aligning the human *KCNJ10* model to the template using PyMOL. PyMOL was also used to analyse and visualise hydrogen and disulfide bonds. Refined 3D models (wild‐type and mutated) were assessed for quality using Ramachandran plot analysis. The stability of the mutant proteins compared to the wild‐type was evaluated by measuring ΔΔG using ICM‐pro (Molsoft LLC).

## 3 Results

### 3.1 Clinical Findings

**FIGURE 1:**
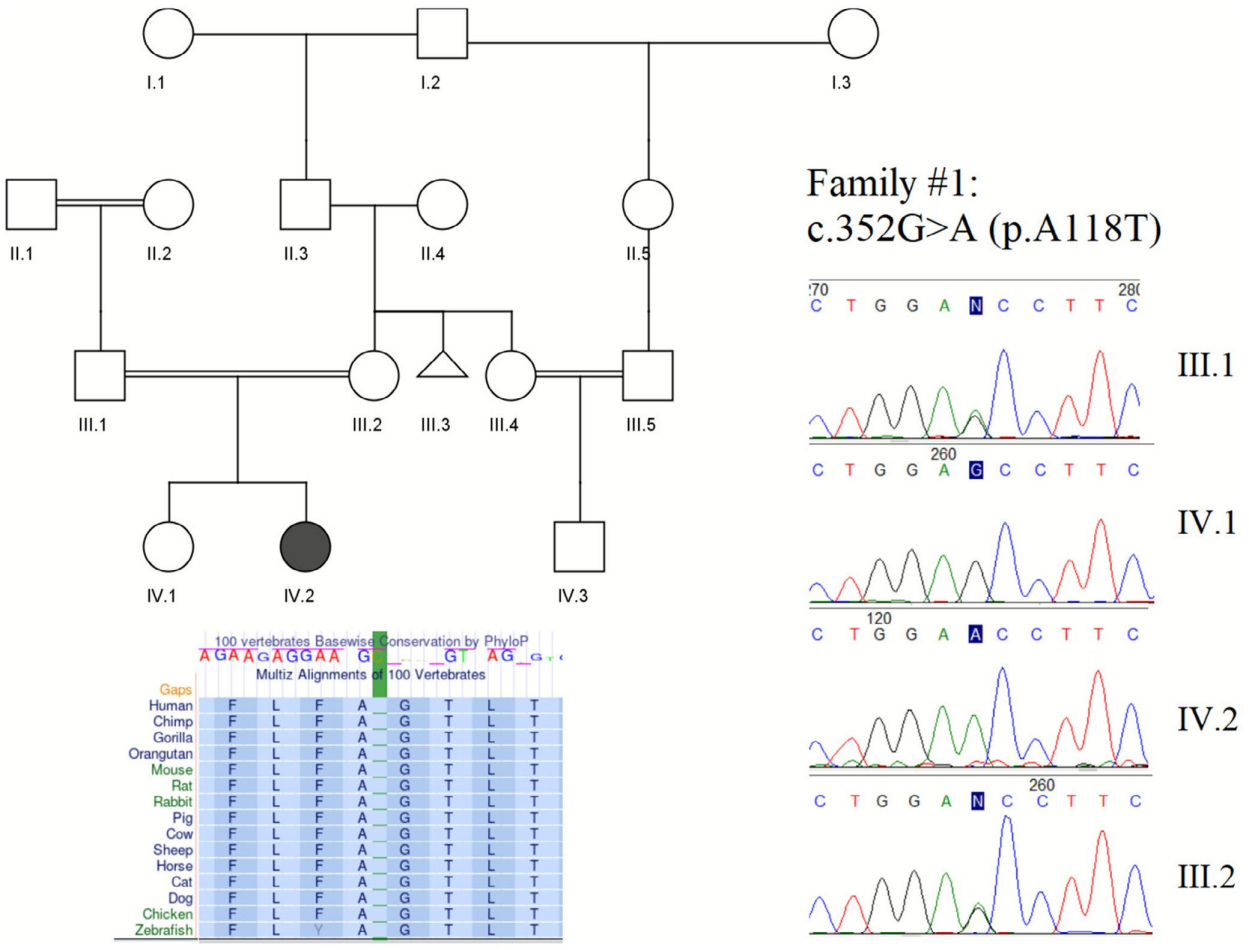
Pedigree and sanger sequencing data of the family 1. Conservation status of the amino acid is also provided.

In Family 1, the proband was a 2‐year‐old girl of Lur (southern Iran) ethnicity. Although there was no known family history of neurological illnesses, one cousin was found to have hearing impairments (Figure 1). The proband was diagnosed with bilateral nystagmus, hearing impairments, developmental delay and intellectual disabilities. She also faced difficulties in speech development, was unable to sit or walk and had issues with coordination, balance and ataxia. Additionally, she began experiencing seizures at the age of 3 months. Laboratory tests indicated normal amounts of urea, creatinine, sodium, potassium and calcium. No PKD‐related symptoms were reported in healthy individuals of the family.

**FIGURE 2:**
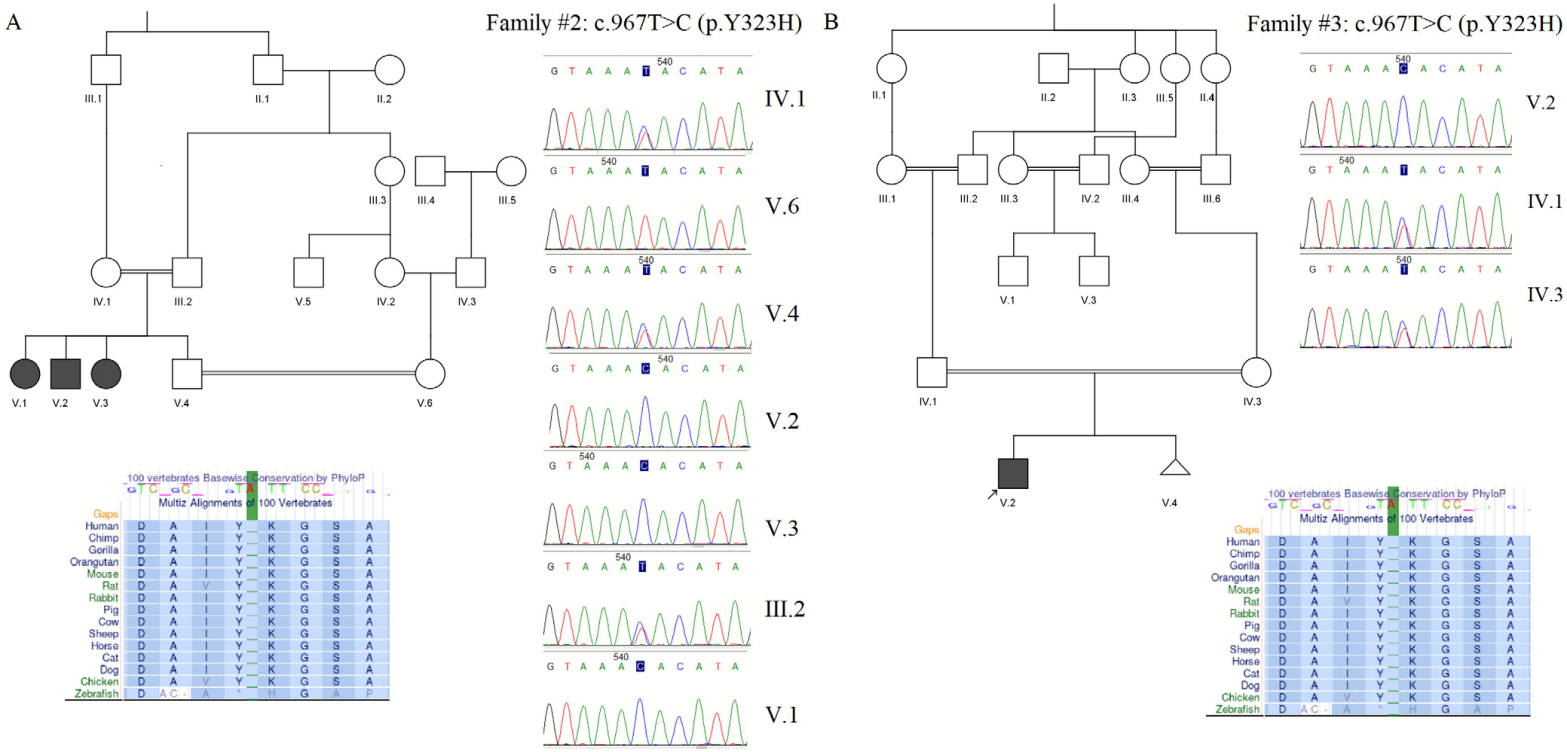
Pedigree and sanger sequencing data of the family 1. Conservation status of the amino acid is also provided.

Family 2 was a consanguineous Persian ethnic family from southern Iran. The family had four children—two boys and two girls. Three children (aged 10, 17 and 23 years old, with the proband being 23) exhibited similar neurological symptoms (Figure 2A). Seizures began occurring when they were 3 months old. In addition to experiencing ataxia, the affected individuals had difficulty walking. They were able to hear well but had trouble with speech development. Due to intellectual disabilities, all affected individuals attended a special‐needs school. Blood evaluation indicated the urea, creatinine, sodium, potassium and calcium levels in normal range. Additionally, there was one other relative with an intellectual handicap, but no other neurological abnormalities or PKD in the pedigree.

Family 3, originating from south Iran, included a 1.5‐year‐old boy proband, resulting from a consanguineous marriage. The child could not walk or sit and had difficulty communicating (Figure 2B). Seizure was reported at the age of 3 months. WES was requested due to neurological abnormalities in two additional relatives. The onset of seizures in these relatives occurred at the age of eight. Despite reaching early developmental milestones, they later experienced a deterioration in motor skills, particularly in walking, showing signs of ataxia. Intellectual impairment became increasingly evident as they aged. Also, no abnormal urea, creatinine, sodium, potassium and calcium amounts were mentioned in blood analysis. No hearing defects or PKD were reported in this family.

### 3.2 Genetic Analysis

A stepwise filtration process based on frequency, genomic location, inheritance mode and HPO term‐related genes revealed 10–15 putative causal variants for each family. All tolerable or benign entries were excluded using in silico prediction algorithms and previously provided clinical data. Further precise evaluation and scoring of the pathogenicity of the putative variants, based on the aforementioned in silico predictive tools, identified potential causative variations in the *KCNJ10* gene in each proband.

In Family 1, a novel variant, *NM_002241.5:c.352G>A, p.A118T*, was identified in the second exon of *KCNJ10* as a candidate causal mutation in the homozygous state. The proband's characteristics and inheritance pattern closely aligned with knowledge of SeSAME syndrome caused by *KCNJ10* mutations. According to MutationTaster, this variant is predicted to be disease‐causing and the phastCons conservation score of 1.0 indicates a high likelihood of evolutionary conservation across 46 distinct species. Additionally, phyloP reported a score of 7.898 (with 10 representing the maximum level of conservation).

This variant was shown to have a deleterious effect on protein function, as indicated by a CADD score of 27.3 and a MetaRNN score of 0.9679. It was classified as a Variant of Uncertain Significance (VUS) according to ACMG criteria and was absent from public variant databases, including GnomAD, 1000 Genomes and ClinVar. PCR‐Sanger sequencing subsequently confirmed the homozygous state of the *KCNJ10:c.352G>A* variant in the proband and the heterozygous state in her parents, suggesting paternal and maternal inheritance.

In Families 2 and 3, another variant, *NM_002241.5:c.967 T>C, p.Y323H*, was identified as a candidate causal mutation in the probands. This mutation affects the *KCNJ10* gene at exon 2. MutationTaster predicted this variation to be disease‐causing, with CADD and MetaRNN scores of 27.4 and 0.985, respectively. With a phyloP score of 7.568, the amino acid at this location is considered highly conserved. This variation was previously reported as a VUS in the ClinVar database in another Iranian family and was evaluated as ‘likely pathogenic’ according to ACMG criteria. Furthermore, it was absent from population frequency databases. Sanger sequencing confirmed the presence of the homozygous mutation in other affected individuals and the heterozygous carrier state in all parents. Table 1 summarises information regarding each variant.

**TABLE 1:** Summary of the information regarding variants discovered in the study.

Family #	Variant	MetaRNN	CADD	PhyloP100way	SIFT	PolyPhen2	Mutation‐taster	ACMG/InterVar evidences
1	NM_002241.5:c.352G>A	0.968	27.3	7.905	Deleterious	Probably damaging	disease causing	VUS (PM2, PP3)
2&3	NM_002241.5:c.967 T>C	0.985	26.2	7.568	Deleterious	Probably damaging	disease causing	Likely pathogenic (PP3, PM2)

### 3.3 Homology Modelling

Using Phyre2, 310 residues of *KCNJ10* (82% of the sequence) were modelled with 100.0% confidence based on the single highest‐scoring template. Ramachandran plots were employed to assess the reliability of the refined structures following optimisation with ModRefiner. The projected models demonstrated high accuracy, as evidenced by the percentage of residues in favoured (91.4%), allowed (8.3%) and outlier (0.3%) conformations.

**FIGURE 3:**
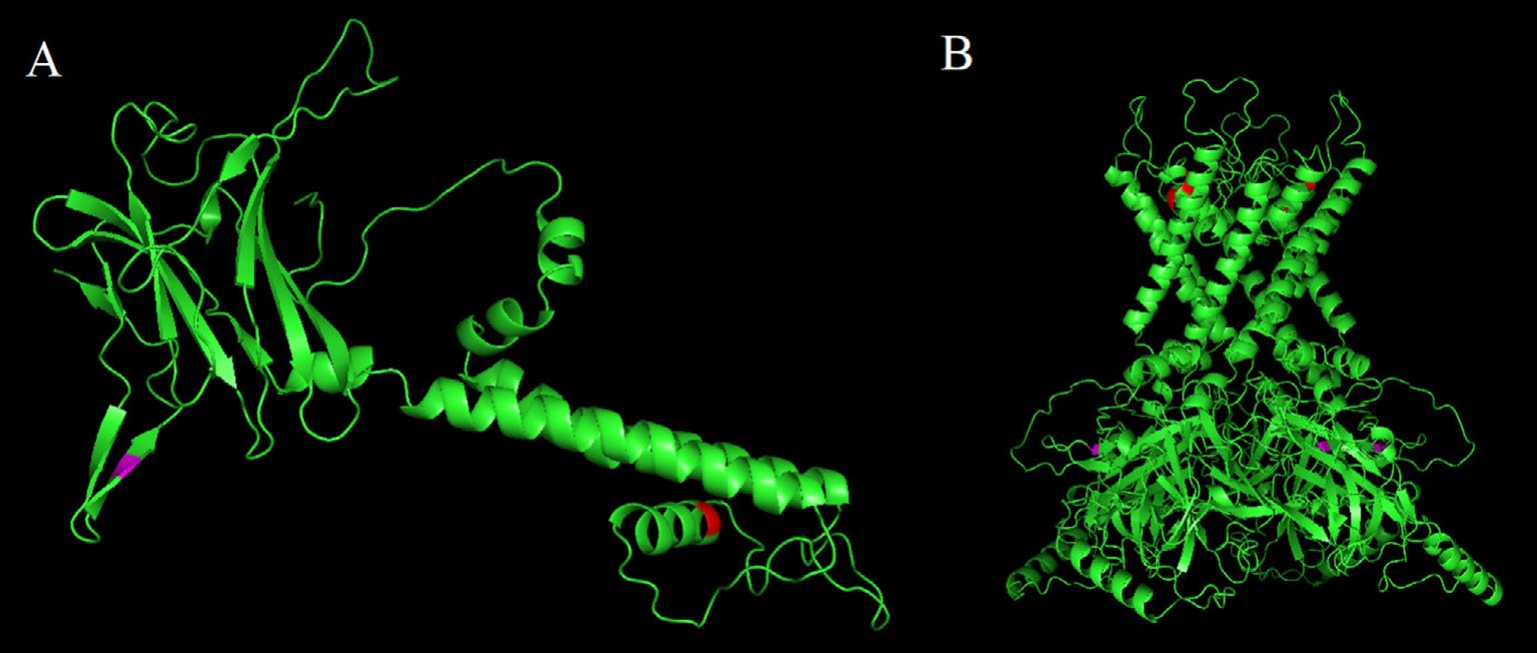
Predicted 3D model of monomeric KCNJ10 protein (A) and tetrameric Kir4.1 (B). Amino acids 118 and 323 are shown in red and magenta respectively.

Subsequently, rat Kir4.1 in complex with PIP_2_ and Lys05 was retrieved from the Protein Data Bank (PDB accession: 8I5M) and the rat homologue was used as a template to construct a homotetrameric structure. PyMOL software was utilised to visualise the predicted structures (Figure 3A,B).

**FIGURE 4:**
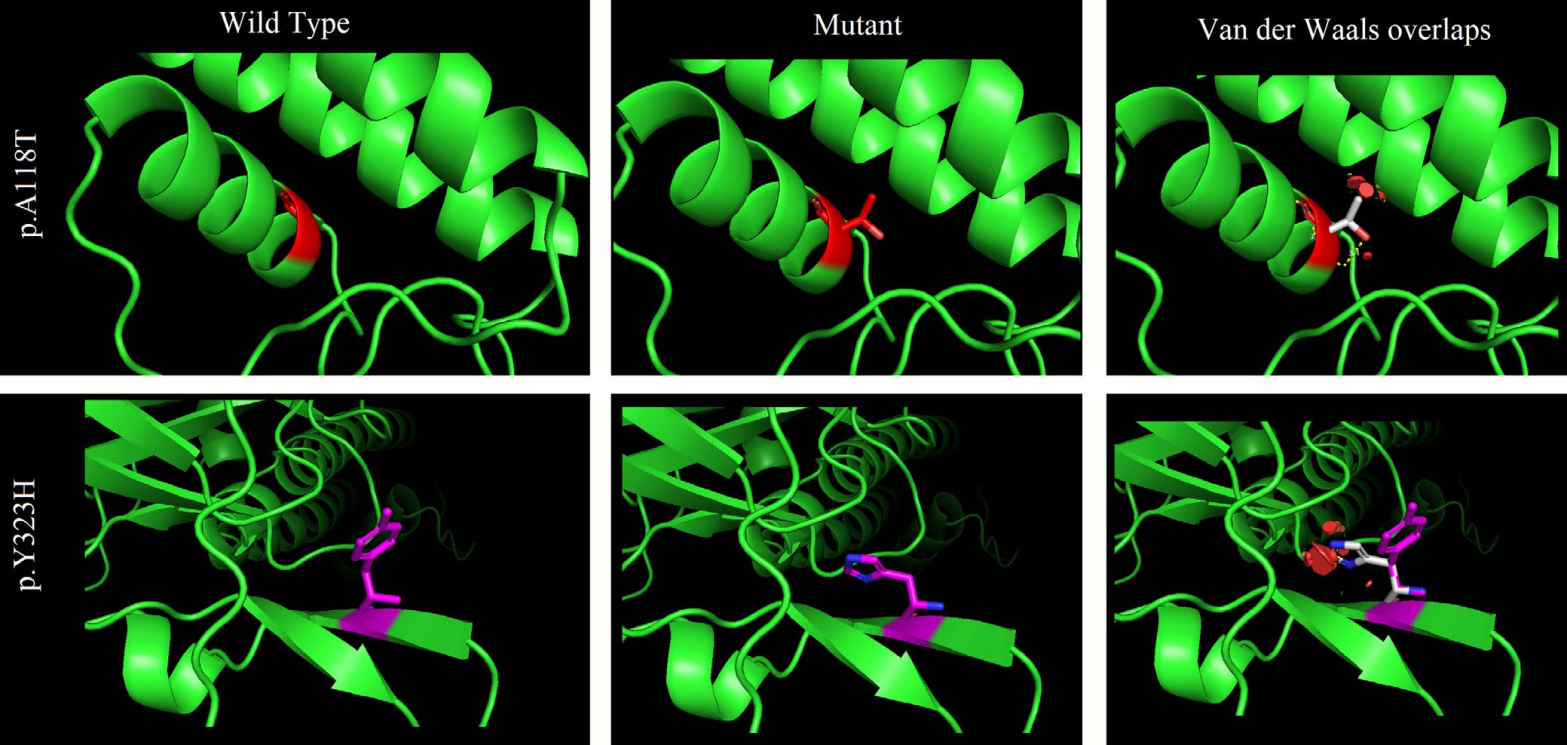
Wild type and mutant amino acids are predicted for each variant. The most frequent occurrence of rotamers was selected, 83.3% for p.A118T and 25.6% for p.Y323H. Red disks in the right panel indicate significant van der Waals overlaps.

As the decrease in protein stability is linked to a positive change in Gibbs free energy, the presence of the p.A118T variant (Family 1) reduced the structural stability of the human ATP8A2 protein, with a free energy change (ΔG_WT_—ΔG_Mutatnt_) calculated by ICM as 0.5586 Kcal/mol. Similarly, the p.Y323H variation (Families 2 and 3) significantly reduced the protein's structural stability, with a calculated free energy change of 0.9162 kcal/mol (ΔG_WT_—ΔG_Mutatnt_) (Figure 4).

## 4 Discussion

In this study, we identified two novel homozygous missense variants in the *KCNJ10* gene in three unrelated families: c.352G>A (p.A118T) in family 1 and c.967T>C (p.Y323H) in Families 2 and 3. The phenotypes observed in these families were consistent with previously reported cases of this syndrome. In certain brain regions, Kir4.1 channels are expressed as homomers, while in others, they form heteromeric channels with Kir5.1 (Hibino et al. 2004). This distinction might account for the observed clinical variability among the patients. Furthermore, according to the identified mutation, tetramerization with Kir5.1 might be influenced, which potentially contributes to the phenotypic differences.

According to the HGMD database, 51 unique mutations in the *KCNJ10* gene have been documented so far, many of which were summarised in a recent study (Lo et al. 2022). The most prevalent phenotype among patients is epilepsy, caused by disturbances in brain potassium homeostasis (Butt and Kalsi 2006; Haj‐Yasein et al. 2011). Ataxia, the second most common phenotype, is characterised by symptoms such as tremors and an unstable gait due to cerebellar impairment (Abdelhadi et al. 2016; Morin et al. 2020). Sensorineural deafness, which is linked to disrupted potassium recycling in the inner ear (Zdebik et al. 2009), along with renal tubulopathy manifesting as hypokalaemia, hypomagnesaemia and metabolic alkalosis, are also frequently observed phenotypes (Thompson et al. 2011). Moreover, atypical characteristics of EAST syndrome, including autism spectrum disorder (Sicca et al. 2016; Cheng et al. 2021) and white matter abnormalities (Al Dhaibani et al. 2018; Severino et al. 2018), have been noted in the cases described.

Several in silico methods suggest that the KCNJ10 mutation p.A118T is deleterious. Substituting the non‐polar, aliphatic and hydrophobic alanine residue with a polar, uncharged, hydrophilic arginine residue may alter the stability of the protein structure and subsequently affect the protein‐folding mechanism. According to InterPro, the variant is located in the inwardly rectifying transmembrane domain of the protein, which accommodates around 66% of the gene's pathogenic/likely pathogenic mutations based on the ClinVar database. Cells with KCNJ10 mutations in this domain were more hyperpolarised than mock‐transfected cells but not as much as WT KCNJ10‐expressing cells (Reichold et al. 2010). Additionally, homology modeling indicated reduced protein stability due to the p.A118T mutation.

p.Y323H in KCNJ10 is also considered a harmful mutation according to in silico prediction methods. Substituting a polar residue (HIS) with a largely non‐polar residue (TYR) in the protein structure of KCNJ10 may affect the interactions of neighbouring residues or substrates, which could influence protein folding and function. Furthermore, switching from a sizeable aromatic side chain to a smaller imidazole ring may impact the structure and stability of the protein. This mutation affects the cytoplasmic C‐terminal domain of the Kir4.1 protein. Based on earlier studies, this domain is essential for both the proper intracellular localisation of Kir4.1 protein (Tanemoto et al. 2004) and its PKC‐mediated regulation (Rojas et al. 2007). The loss of developmental milestones observed in Family 3 was not previously documented for SeSAME syndrome and it is possible that the protracted seizures, rather than the KCNJ10 mutation, caused brain hypoxia.

Here, the p.Y323H mutation was detected in two distinct families. Additionally, it was independently submitted in 2020 in another unrelated Iranian family to the ClinVar database (https://www.ncbi.nlm.nih.gov/clinvar). This may indicate its high prevalence in the Iranian population. According to our literature review, many reported mutations are from the Middle East and Western Asia. For example, p.R65P is the most commonly reported mutation in the KCNJ10 gene, observed in Pakistani families (Table 2). A founder effect could explain this observed distribution as the families have the same ethnicity. However, further research is needed to validate these findings, as the prevalence of these mutations may be influenced by underreporting or insufficient genetic screening in other regions.

**TABLE 2:** Summary of the variants previously reported in middle east/western Asia.

Variant	Ethnicity	Consanguinity	Seizure (age of onset in month)	Sensorial deafness	Ataxia	Intellectual disability	Electrolyte imbalance	Ref
T164I	Afghan	Yes	Generalized (3)	Progressive hearing loss at age of 18	Yes	Yes	persistent hypokalemia, metabolic alkalosis, hypomagnesemia and low Ca^2+^/creatinine ratio	(Scholl et al. 2009)
C140R	Turkey	Yes	Yes (4)	Yes	Yes	Yes	Yes	(Scholl et al. 2009)
I60T	Arabic	Yes	Yes (3)	Not tested	Yes	Yes	No	(Siepel et al. 2005)
R297C	Iranian	Yes	Yes (2)	Yes	Yes + Tremor	No	Hypomagnesaemia. Hypokalaemia	(Zdebik et al. 2009)
R65P	Pakistani	Yes	Yes (4)	Yes	Yes	No	Hypokalaemic metabolic alkalosis, Hypomagnesaemia with renal magnesium wasting	(Zdebik et al. 2009)
G77R	Arabic	Yes	Yes (3)	Yes	Yes + Tremor	No	Hypokalemic metabolic alkalosis. Hypomagnesaemia. Hypocalciuria	(Bockenhauer et al. 2009)
L218F*(KCNJ10)*/G288S*(KCNT1)*	Saudi Arabian	No	Yes (3)	No		No	Low phosphate and urea	(Thompson et al. 2011)
R175Q	Iranian	Yes	Yes (infancy)	Yes	Yes		Hypomagnesaemia. Hypokalaemia	(Reichold et al. 2010)
A118T	Iranian	Yes	Yes (3)	Yes	Yes	Yes		This paper
Y323H	Iranian	Yes	Yes (3)	No	Yes	Yes		This paper

Regarding intellectual disability, it is essential to note that children with SeSAME syndrome are very challenging to evaluate due to the severity of other neurological symptoms (Abdelhadi et al. 2016). Particularly in the early years, decreased speech and reduced general intellectual development are likely caused by severe ataxia, seizures and sensorineural hearing loss. When provided with the appropriate care, some children with functionally disruptive mutations in KCNJ10 can attend regular schools. Mainly, if symptoms are detected early, it appears that intellectual impairment may be secondary to the other types of disability encountered by these individuals. However, the families in our research did not have access to this kind of care due to socioeconomic constraints. Our patients showed no signs of renal involvement, which was also noted in prior research, suggesting that co‐expression with Kir5.1 may restore the functional deficiency in these cases (Al Dhaibani et al. 2018; Nicita et al. 2018).

To sum up, our research uncovers two new KCNJ10 mutations in three unrelated Iranian families, contributing to expanding knowledge about the genetic causes of SeSAME syndrome. One of these mutations, p.Y323H, is located in the Kir4.1 channel's cytoplasmic C‐terminal domain (CTD), where harmful mutations are rarely found. While further study is required to validate this, it suggests that the CTD may play a more significant role in disease aetiology than previously thought. According to our in silico analysis, this mutation could affect the stability and function of the protein. However, lack of functional validation studies such as patch‐clamp electrophysiology, enzyme activity assays, or immunocytochemistry tests is the major limitation of our study. The lack of renal involvement in the affected individuals may be possibly explained by the compensatory mechanisms, such as co‐expression with Kir5.1, but this remains speculative. These findings underscore the importance of continued genetic research to clarify the molecular causes and clinical heterogeneity of SeSAME syndrome.

## Author Contributions

S.S., F.S., M.M. and S.M. participated in data curation, original draft writing, review and editing. S.S. performed the WES analysis. M.E. had the role of conceptualization, supervision and project administration.

## Funding

The authors have nothing to report.

## Ethics Statement

The Shiraz University of Medical Sciences Ethical Board gave its approval for this work under IR.SUMS.REC.1402.154 code.

## Consent

Written informed consent was obtained from all families included in the study.

## Conflicts of Interest

The authors declare no conflicts of interest.

## Data Availability

The data that support the findings of this study are available on request from the corresponding author. The data are not publicly available due to privacy or ethical restrictions.
